# FDG-PET in suspected dementia with Lewy bodies: a case report

**DOI:** 10.1186/s12877-019-1166-3

**Published:** 2019-05-28

**Authors:** Astrid Melani Suantio, Hian Liang Huang, Cecilia Sze Nga Kwok, Darren Cheng Han Teo, Minh Ha Nguyen

**Affiliations:** 1 Department of Internal Medicine, Geriatric Medicine, Sengkang General Hospital, E Way, Sengkang, 544886 Singapore; 2 Department of Nuclear Medicine and Molecular Imaging, Singapore General Hospital, Outram Road, Singapore, 169608 Singapore; 3 Department of Psychiatry, Singapore General Hospital, Outram Road, Singapore, 169608 Singapore; 4 Singhealth Residency Program, MOH Holdings Pte Ltd, Harbour Front, Singapore, 099253 Singapore; 5 Department of Geriatric Medicine, Singapore General Hospital, Outram Road, Singapore, 169608 Singapore

**Keywords:** Dementia with Lewy bodies, FDG-PET, Imaging, Rivastigmine

## Abstract

**Background:**

Dementia with Lewy bodies (DLB) is still underdiagnosed or mistaken for other types of neurodegenerative diseases. Biomarkers such as 18-Fluorodeoxyglucose Positron Emission Tomography (FDG-PET) can be helpful.

**Case presentation:**

A 72-year-old gentleman presented with postural hypotension, hallucination, Parkinsonism and recurrent falls. He also had rapidly progressing cognitive impairment. CT and MRI brain showed atrophy of the frontal lobes with preservation of the hippocampi. FDG-PET was suggestive of DLB. He was subsequently treated with Rivastigmine, with significant improvement of his symptoms.

**Conclusion:**

This case highlights the challenges in diagnosis of an elderly patient with DLB, the use of neuro-imaging as a diagnostic biomarker, the avoidance of the use of antipsychotic and the response to pharmacological treatment with Rivastigmine after a probable diagnosis of DLB.

## Background

Dementia with Lewy bodies (DLB) is the second most common type of degenerative dementia, accounting for approximately 4.2% of all diagnosed dementia in the community and up to 7.5% of those in secondary care [1]. Lewy body protein was first discovered by a German scientist, Friederich H. Lewy, in the early 1900s, while doing research on Parkinson’s disease [2]. These abnormal protein deposits disrupt normal function of the brain and affect the patient’s cognition, behavior, movement as well as their body’s autonomic function [2]. Lewy body protein is found in several neurodegenerative diseases, including DLB, Parkinson’s disease and Parkinson disease dementia (PDD) [3].

Clinical diagnosis of DLB still remains challenging as there is considerable overlap in the early clinical presentations between DLB and both Alzheimer disease (AD) and Parkinson’s disease. The characteristic clinical features of DLB include fluctuating cognition, spontaneous Parkinsonism, recurrent visual hallucinations, rapid eye movement (REM) sleep behavior disorder, severe sensitivity to antipsychotic agents and severe autonomic dysfunction. In 2017, the fourth consensus report of the DLB Consortium has refined its recommendation on the clinical and pathological diagnosis of DLB. These revised diagnosis criteria now distinguish clearly between clinical features and diagnostic biomarkers [4]. Clinical signs and symptoms are featured as core or supportive, in which the presence of rapid eye movement (REM) sleep behavior disorder is now one of the core features instead of supportive feature in the previous report. Similarly, biomarkers are also divided up into indicative and supportive categories with greater weight on iodine123 - metaiodobenzylguanidine (MIBG) myocardial scintigraphy (Table 1).

**Table 1:** Revised criteria for the clinical diagnosis of Dementia with Lewy bodies (DLB)

Central feature (essential for a diagnosis of possible or probable DLB) • Dementia defined as progressive cognitive decline of sufficient magnitude to interfere with normal social or occupational function • Prominent or persistent memory impairment may not necessarily occur in the early stages but is usually evident with progression • Deficits on tests of attention, executive function and visuospatial ability may be especially prominent Core features (2 core features or 1 core feature with 1 or more indicative biomarkers for a diagnosis of probable DLB; 1 core feature for possible DLB) • Fluctuating cognition with pronounced variation in attention and alertness. • Recurrent visual hallucinations that are typically well formed and detailed • REM sleep behavior disorder, which may precede cognitive decline • Spontaneous features of parkinsonism Supportive features • Severe neuroleptic sensitivity • Postural instability • Severe autonomic dysfunction • Hallucinations in other modalities, delusions, apathy, anxiety, and depression Indicative biomarkers (if 1 or more indicative biomarkers is present but there is no core clinical features, possible DLB can be made) • Reduced dopamine transporter uptake in basal ganglia demonstrated by SPECT or PET • Abnormal (low uptake) iodine123 - metaiodobenzyguanidine (MIBG) myocardial scintigraphy • Polysomnographic confirmation of REM sleep without atonia Supportive biomarkers • Relative preservation of medial temporal lobe structures on CT/MRI scan • Generalized low uptake on SPECT/PET perfusion/metabolism scan with reduced occipital activity +/− the cingulated island sign on FDG-PET imaging • Prominent posterior slow wave activity on EEG with periodic fluctuations in the pre-alpha/theta range *From McKeith IG, Boeve BF, Dickson DW* et al. *Diagnosis and management of dementia with Lewy bodies: Fourth consensus report of the DLB Consortium. Neurology. 2017; 89(1): 88–100.*	

Compared to AD, DLB has less favorable prognosis with accelerated cognitive decline, shorter life span, and increased risk of institutionalization and mortality [5, 6]. The prevalence of DLB in Singapore has not been studied extensively, however the prevalence of dementia in Singapore is reported to be 2.6% [7]. The management of patients with DLB is usually complex, requiring a comprehensive treatment program with multidisciplinary approach, as well as care-giver education and support [4]. Although there is limited evidence to support a particular treatment and none can alter the course of the disease, a combination of non-pharmacological and pharmacological approaches has been shown to improve the patient’s quality of life [8].

This case report illustrated the challenge in clinical diagnosis of the patient with DLB, the use of neuro-imaging as a diagnostic biomarker and the patient’s response to pharmacological treatment after a probable diagnosis of DLB was made.

## Case presentation

Our patient was a 72 year- old Chinese gentleman with background history of type 2 Diabetes Mellitus and Hypertension. He was single, staying alone and an ‘A’ level graduate. He first presented to a neurology clinic in September 2017 for bradykinesia and monotonous speech, associated with cogwheeling and lead-pipe rigidity in bilateral upper limbs on clinical examination. There was no tremor noted. At this time, the impression by his neurologist was possible Parkinson’s disease. After prescription of Madopar, his symptoms and signs did not improve. In November 2017, he presented to the emergency department for hypotension (blood pressure 85/48) and recurrent falls. Further cognitive history from his sister, who visited daily, revealed that he had been experiencing forgetfulness, disorientation in time and place and difficulty expressing himself for more than 5 months. These cognitive symptoms fluctuated during the course of the day. Over the past few months, our patient became increasingly paranoid. He suspected his sister and neighbors of stealing money from his bank, changing the locks in the house. He believed the neighbors were talking about him and climbing into his house to take his things, and reported seeing his deceased parents. More recently, his sister observed unusual behaviors such as walking around the house naked and eating food off the floor. As a consequence, he was no longer able to take care of his own instrumental activities of daily living (iADL) as well as his basic activities of daily living (bADL). He became more dependent on his family member and had to move in and stay with his sister for 2 months before his hospitalization. Patient’s sister also noted that his cognitive impairment predated his Parkinsonism features, together with episodes of recurrent falls and a previous episode of loss of consciousness.

On physical examination, patient had severe postural hypotension, with significant systolic blood pressure drop of 40–70 mmHg after 3 min. Neurological examinations found bilateral upper limbs rigidity and cogwheeling as well as shuffling gait. During his hospital stay, patient was noted to have poor sleep at night and kept pacing up and down in the ward. Both his subjective and objective assessment showed that he fulfilled criteria for major neurocognitive disorder (Table 2).

**Table 2:** Objective measurement on patient’s cognition

Measurements applied	Score
• MMSE	20/30
- Orientation	7/10
- Immediate recall	3/3
- Attention	2/5
- Delayed recall	2/3
- Language	6/8
- Construction	0/1
• GDS	3/20
• CLOX	
- CLOX 1	5/15
- CLOX 2	5/15

*MMSE* mini mental state examination, *GDS* geriatric depression scale, *CLOX* an executive clock drawing test

Further evaluation at the hospital revealed unremarkable laboratory investigations for any other causes of rapidly progressive dementia. There was no suggestion of any autoimmune, metabolic or infective causes. A transthoracic two dimensional echocardiogram (2DEcho) showed no valvular lesions with normal left ventricular systolic function. Electrocardiogram (ECG) showed normal sinus rhythm. Furthermore, patient had an autonomic nervous system study which showed positive sudomotor dysautonomia**.**

**Fig. 1:**
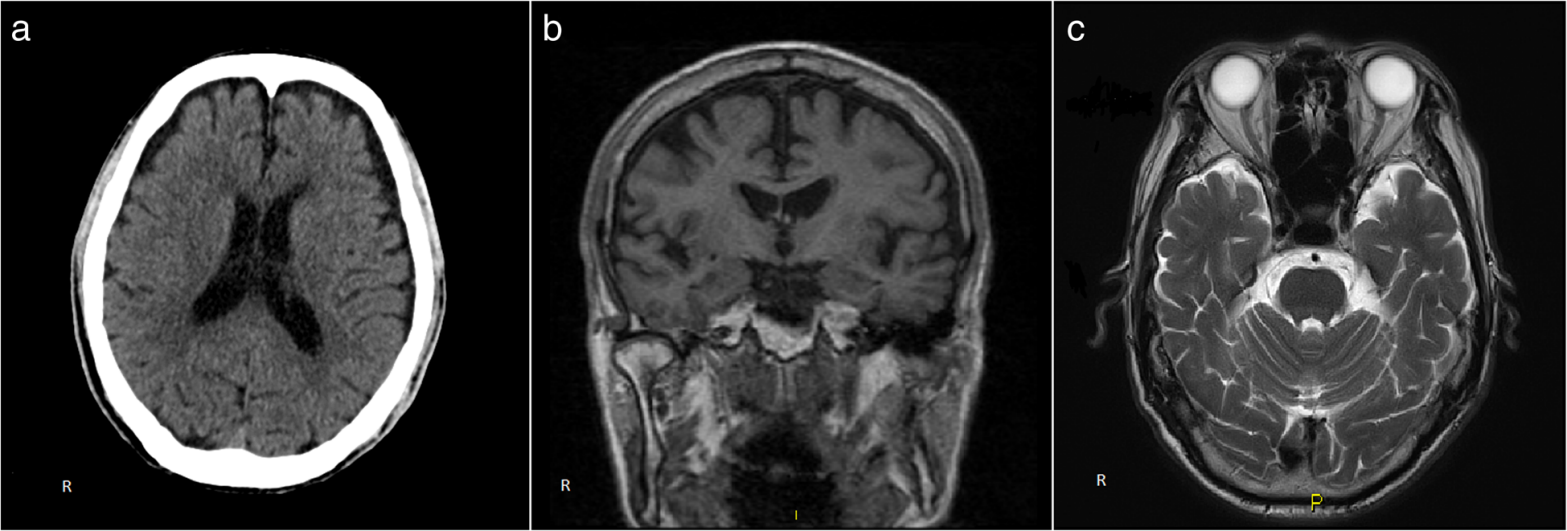
**a** Axial CT brain of our patient showed atrophy of bilateral frontal lobes (**b**) T1 weighted coronal MRI brain showed preservation of the hippocampi (**c**) T2 weighted axial MRI brain showed preservation of bilateral medial temporal lobes

**Fig. 2:**
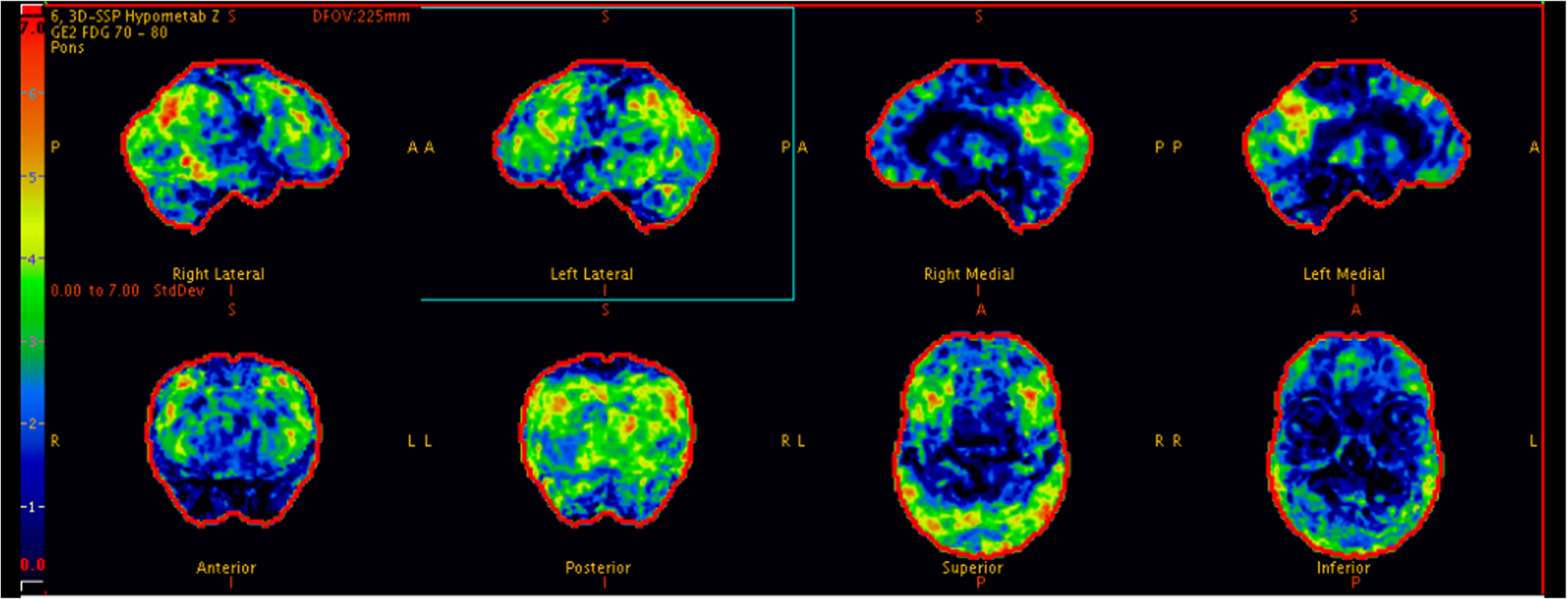
3D Stereotactic Surface Projection of FDG PET of our patient showed hypometabolism in bilateral occipital lobes, posterior parietal lobes, and to a lesser extent, bilateral frontal lobes. The metabolism in posterior cingulate gyri and bilateral temporal lobes is preserved. These findings were supportive of Dementia with Lewy Body (DLB). Involvement of the frontal lobes goes against this being a Posterior Cortical Atrophy variant of Alzheimer’s dementia

Initial CT scan of the brain showed marked bi-frontal atrophy. MRI of the brain reported as mild-moderate midbrain volume loss and symmetrical bilateral hippocampi without disproportionate volume loss (Fig. 1). At this stage, we considered differentials of Lewy Body dementia, Frontotemporal dementia, or Alzheimer’s disease. Subsequently, an 18-Fluorodeoxyglucose Positron Emission Tomography scan (FDG-PET) of the brain was performed (GE Discovery 690, Milwaukee, USA). It showed hypometabolism of bilateral occipital, posterior parietal lobe and to a lesser extent, bilateral frontal lobes with relative sparing of the posterior cingulate gyrus and bilateral temporal lobes (Fig. 2). These imaging and clinical findings are supportive of a probable diagnosis of Dementia with Lewy Body (DLB).

Patient was started on Rivastigmine patch for DLB as well as fludrocortisone acetate and midodrine for his postural hypotension. After one month of starting these medications, patient showed improvement in his cognition and hallucination. His postural hypotension improved significantly with only a single dose of midodrine daily and he was reported to sleep well at night.

## Discussion and conclusions

Our patient presented as a typical case of an elderly man with a fall admitted to a medical ward. Without elucidating patient’s cognitive history from a reliable source, the diagnosis of a major neurocognitive disorder may have been missed entirely. In a relatively short period of 5 months, the patient’s cognition, his bADL and iADL had worsened significantly. Although Parkinson’s disease patients may eventually develop dementia and hallucination during the course of their disease, these symptoms do not usually occur early [9]. Our patient presented with several core and supporting features of DLB namely dementia, Parkinsonism, vivid hallucination, postural instability and severe autonomic dysfunction and his symptoms progressed in a short time frame. Furthermore, as the patient had prominent psychotic symptoms which caused significant care giver stress, anti-psychotic was considered initially as a treatment option. However, DLB patients are known to have neuroleptic hypersensitivity, which can result in death in the worst-case scenario [4]. The treatment of behavioral disturbances in DLB is not, therefore, antipsychotic. Instead, Rivastigmine, an acetylcholinesterase inhibitor, has been shown to improve patient’s activities of daily living, cognitive function and behavioral disturbances [10]. This highlights the importance of clinching the diagnosis of DLB using all means available. Since our patient’s CT and MRI were not conclusive of DLB diagnosis, we proceeded with FDG-PET imaging.

**Fig. 3:**
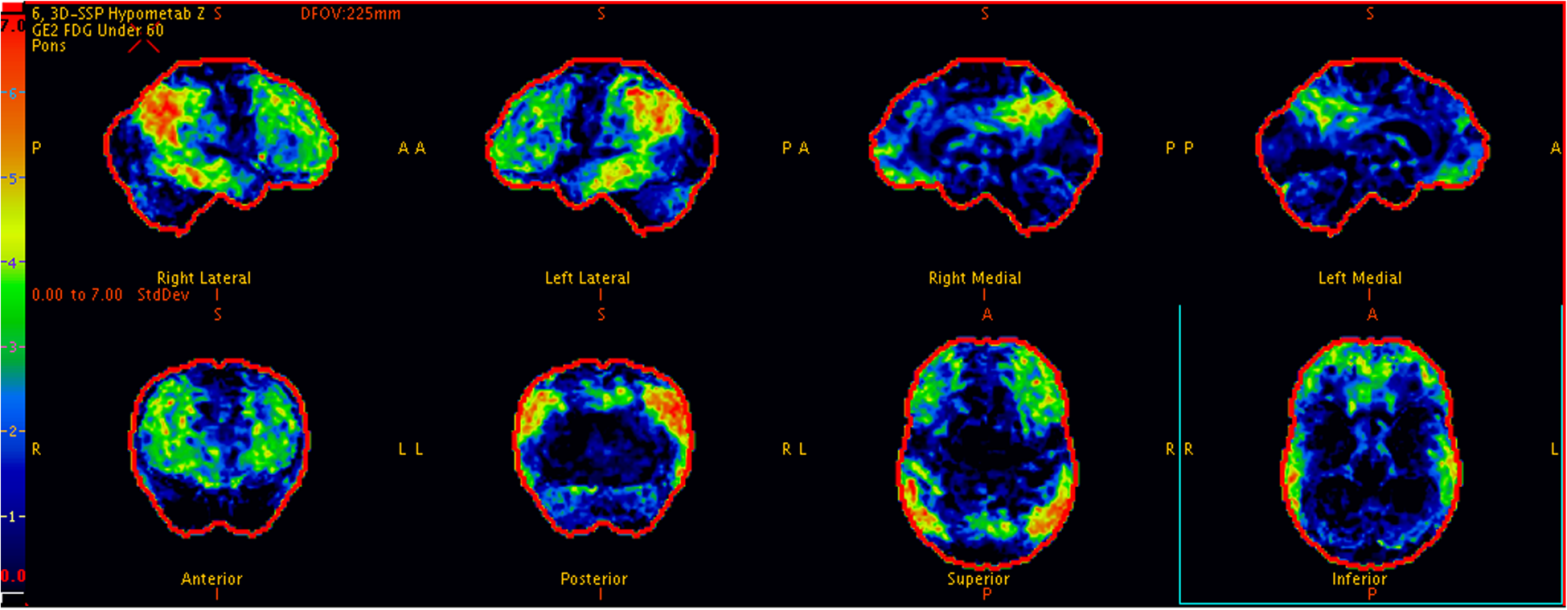
In contrast to Fig. 2, 3d Stereotactic Surface Projection of FDG PET with typical findings of Alzheimer’s disease showing hypometabolism of the bilateral inferolateral temporal lobes, bilateral precuneus, posterior cingulate and angular gyri (parietal lobes). There is relative sparing of the occipital lobes and involvement of the posterior cingulate gyri

In Singapore public hospitals, FDG-PET is not routinely used for investigation of rapidly progressive dementia yet. Imaging is however recommended as part of the investigations of people with suspected dementia in UK, European, and US guidelines [11]. Imaging is now also embedded in several modern diagnostic criteria for different dementias, including AD and DLB. In the FDG-PET of a normal elderly, the glucose metabolism is higher in posterior cingulate cortex as compared to other parts of the brain [12]. In AD, there is hypometabolism in the parietotemporal association cortices, posterior cingulate and precuneous regions with spread to the frontal cortex with progression of disease. There is also relative preservation of the basal ganglia, primary sensorimotor cortices, visual cortices and cerebellum (Fig. 3). The hypometabolic regions in patients with DLB are similar to those in AD but the relative metabolic reduction is more severe in the occipital cortices and less severe in the medial temporal lobes. Occipital hypometabolism is a key feature of DLB that discriminates it from AD [13]. Interestingly, the involvement of occipital cortices is not solely found in patients with DLB as it is also seen in PD and PDD. This observation could be due to the fact that all three diseases share the same pathology of Lewy bodies spreading in the cerebral cortices [14]. By using FDG-PET, our patient avoided the dangers of antipsychotic use and was treated with Rivastigmine instead which resulted in significant improvement in his cognition and function.

This case demonstrated the difficulty in elucidating dementia with Lewy bodies and the use of FDG-PET as a diagnostic biomarker. A comprehensive assessment, including cognitive history from a reliable informant, thorough physical examination, and cognitive assessments, is essential to differentiate DLB from other types of neuro-degenerative dementia. Biomarkers such as imaging, sleep study, and electroencephalography (EEG) will likely play more important roles in supporting early diagnosis of DLB in the future.

## Data Availability

Not applicable.

## Abbreviations

2DEcho: Two dimensional echocardiogram
AD: Alzheimer’s disease
bADL: Basic activities of daily living
CLOX: An executive clock drawing
CT: Computed tomography scan
DLB: Dementia with Lewy bodies
ECG: Electrocardiogram
EEG: Electroencephalography
FDG-PET: 18Fluorodeoxyglucose Positron Emission Tomography scan
GDS: Geriatric depression scale
iADL: Instrumental activities of daily living
MIBG: Metaiodobenzylguanidine
MMSE: Mini mental state examination
MRI: Magnetic resonance imaging
PDD: Parkinson disease dementia
PET: Positron Emission Tomography
REM: Rapid eye movement
SPECT: Single-photon emission computed tomography
